# Risky sexual practice and associated factors among HIV positive adults attending anti-retroviral treatment clinic at Gondar University Referral Hospital, Northwest Ethiopia

**DOI:** 10.1371/journal.pone.0174267

**Published:** 2017-03-28

**Authors:** Abiyot Abeje Molla, Abebaw Addis Gelagay

**Affiliations:** 1 University of Gondar Hospital, Gondar, Ethiopia; 2 Department of Reproductive Health, College of Medicine and Health Sciences, University of Gondar, Gondar, Ethiopia; National Institute of Health, ITALY

## Abstract

**Introduction:**

Risky sexual practice among people living with HIV/AIDS is a public health concern because of the risk of transmission of the virus to sero-discordant partner/s. There is also the risk of re-infection with new, drug resistant viral strains between sero-concordant partners. However, there is lack of information on risky sexual practices among HIV positive adults. Therefore, this study aimed to assess risky sexual practice and associated factors among adult HIV positive clients at Gondar University Referral Hospital, Northwest Ethiopia, 2015.

**Methods:**

An institution based cross sectional study was conducted at Gondar University Referral Hospital from May to June 2015. A pretested structured questionnaire was used to collect the data. Using systematic random sampling technique, a total of 513 respondents were participated in this study. The data were entered into EPI info version 3.5.3 and transferred to SPSS version 20 for analysis. Descriptive, bivariate and multivariate analyses were done. A P-value <0.05 was considered to determine the statistical significance of the association between factors (independent variables) and risky sexual practice. The Odds ratio was also used to determine the presence and the degree of association between the dependent and independent variables.

**Results:**

A total of 513 respondents were participated in this study. The prevalence of risky sexual practices in the past three months was 38% (95% CI: 33.3%, 42.3%). Being in the age range of 18–29 years (AOR = 2.63, 95% CI: 1.55, 4.47) and 30–39 years (AOR = 2.29, 95% CI: 1.48, 3.53), being single (AOR = 6.32, 95%CI: 2.43, 16.44),being married (AOR = 6.06, 95% CI: 2.81, 13.07), having no child (AOR = 2.58, 95% CI: 1.17, 5.72), and a CD4 count of greater than 500/mm^3^ were factors significantly associated with risky sexual practices.

**Conclusions:**

A considerable number of HIV positive clients had risky sexual practices. It is strongly recommended that the Regional Health Bureau and health service providers working at Gondar University Hospital especially in the ART Clinic need to work hard on behavioral change communication.

## Introduction

The emergence and increased access to highly active antiretroviral therapy (HAART) and the presence of an unacceptably high number of new HIV infection resulted in a large number of people living with the virus. Globally, an estimated 36.9 million people were living with HIV, while 2 million were newly infected in 2014. The largest number (1.4 million) of new HIV infections were estimated to be in Sub Saharan Africa[1]. The vast majority of people living with HIV are in low- and middle-income countries, particularly in sub-Saharan Africa. According to the 2013 report, Sub-Saharan Africa, the hardest hit region, was home to71% (24.7million) of people living with HIV in the world, but it accounts only for about 13% of the world’s population[2].

In Ethiopia, there were an estimated 793,700 people living with HIV and approximately 45,200 AIDS-related deaths in 2013, which has left about 898,400 AIDS orphans. According to the 2011 Ethiopian Demographic and Health Survey (EDHS) report, adult HIV prevalence was estimated at 1.5%. However, HIV prevalence varies with age, sex, and geographical location. Accordingly, the prevalence was almost twice higher among females compared to males (1.9% versus 1.0%, respectively)[3].

A high number of people living with HIV become a potential source of infection[4]. Unless people living with HIV consistently practice safer sex, they can make themselves risky of acquiring other strains of HIV and sexually transmitted infections (STIs), and put others at risk for HIV and other STIs.

Different studies note that after the initiation of highly active antiretroviral therapy (HAART), HIV positive individuals are living longer, becoming healthier, and resuming sexual practice. A follow up study conducted in Uganda noted that sexual activity/practice among HIV positive adults increased from 28% before ART initiation(baseline) to 41% six months after ART initiation. There were also an increased number of sexual partners at six months[5, 6].

A community-based cohort study in Baltimore, Maryland, noted that risky sexual behavior is increased following HAART initiation. Improvement in overall clinical status which may increase interest or ability to engage in risky sexual practice and awareness of the lower risk of transmission while the virus is virologically suppressed by HAART are mentioned as potential reasons for the increased risky sexual behavior[7]. The risk of HIV-transmission to sero-discordant partners and the risk of re-infection with new drug resistant viral strains is a concern for persons practicing risky sexual intercourse[8].

Understanding the risky sexual practice in Ethiopia could have public health importance in that it helps us generate and provide evidence based information to policy makers, program planners and health service providers on the problem which is subsequently essential to design and implement appropriate interventions. However, information about risky sexual practice among HIV positive adults is limited in Ethiopia in general and in the study area in particular. So, this study aimed to determine the prevalence of risky sexual practice and associated factors among HIV positive adults who had follow ups at the ART clinic of Gondar University Referral Hospital.

## Methods

### Study design and period

An institution based cross-sectional study was conducted to assess the prevalence and associated factors of risky sexual practice among HIV positive adults visiting the ART Clinic at Gondar University Referral Hospital from May to June, 2015.

### Study area

The study was conducted at Gondar University Referral Hospital in the chronic HIV care and treatment clinic. Geographically, it is located in Gondar city, Northwest Ethiopia. It is 727 km from Addis Ababa, the capital city of Ethiopia. Gondar University Referral Hospital is currently providing health services to more than 5 million people in the catchment area. ART clinic is one of the outpatient service delivery sites where chronic HIV care and treatment is provided to HIV positive individuals. The clinic started to provide ART services as fee based in 2003 and later, the free based ART service was introduced two years later started. At the moment, 5210 on ART and 2471 pre-ART clients are actively attending their follow-up at the clinic.

### Study population

All HIV positive adults who had follow-up at the ART clinic of Gondar University Referral Hospital during the study period constitute the population of the study.

### Sample size determination and sampling procedure

The sample size was determined using the single population proportion formula by taking the prevalence of risky sexual practice (p) to be 36.9% from a previous study done in Addis Ababa[8] with the following assumptions: 95% confidence level, 5% marginal error. So, the sample size calculated for the first objective was 394 after considering a 10% allowance for non response.

Sample size for factors was computed using a study conducted in Addis Ababa where sex was associated with unprotected sexual practice[8, 9]. A study done in Montgomery identified that alcohol consumption was associated with unprotected sex[10]. By Using EPI info version 7 and taking a 95% confidence level, unexposed to exposed ratio (1:1), and 80% power revealed the following result: n = 389 for sex and n = 471 for alcohol consumption. Therefore, the final sample size was 518 by considering a 10% allowance for non response.

A systematic random sampling technique was used to select the study participants. The sampling interval was determined based on the number of patients who came for follow up to the ART clinic each month. The average number of patients who came to the ART Clinic for follow up in each month was estimated at 2200. By considering monthly client's flow for follow up, the sampling interval was four.

### Operational definitions /Measurement/

- **Risky sexual practice:** having one or more of the following practices during the past three months prior to date of data collection: having multiple sexual partners, casual sex, sex without or inconsistent use of condom even with regular partner, sex with the influence of substance like alcohol.
- **Regular partner:** partner with whom the respondent had regular sexual relationship and perceived by the respondent as spouse or boy/girl friend.

### Data collection tool and procedures

The questionnaire was first prepared in English and translated into the local language (Amharic), and then retranslated to English by language experts to check the consistency. Three data collectors and one supervisor were employed trained on the methods, objectives, and the tool before data collection began. The tool was pre-tested on 25 individuals in the nearby health center, Gondar Poly Health Center. Questions which caused difficulty in the pre-test were rephrased and corrected. The study participants were selected every 4^th^ interval among HIV positive adults who were coming to the hospital during data collection period for routine follow up care and were requested to participate in the study. Before data collection, these participants were given information about the purpose of the study, the possible risks and benefits of participating, the confidentiality of the study, and their right not to participate or withdraw from the study any time. Subsequently, informed consent was taken from each participant and the data were collected using an interviewer administered questionnaire. The supervisor and investigators checked the completeness of the questionnaire daily.

### Data processing and analysis

The collected data were checked for completeness, coded and entered into Epi Info version 3.5.3 and was exported to SPSS version 20 for analysis. Binary logistic regression analysis was made. Each explanatory variable was first analyzed by using bivariate logistic regression and variables with p-values less than 0.2 were further entered in to multivariable logistic regression model to see the independent effect of covariates on risky sexual practice In the multivariable analysis, a p-value less than 0.05 was considered to determine the statistical significance of the association. Crude Odds ratio (COR) in the bivariate analysis and adjusted odds ratio (AOR) in multivariable analysis with 95% CI were used to assess the presence and strength of association between dependent and independent variables.

### Ethical considerations

The Institutional Review Board (IRB) of the University of Gondar, College of Medicine and Health Sciences, Department of Internal Medicine reviewed the full proposal (including the information sheet and consent form/procedure) and approved the study. Additionally, a support letter was obtained from Gondar University Hospital. Respondents were informed about the purpose, procedure, possible risks and benefits of participating, the confidentiality, and their right not to participate or withdraw from the study any time. Subsequently, informed verbal consent was obtained from each participant. Clients, who volunteered to participate in the study were interviewed in private. No name or personal identification was written on the questionnaire.

## Results

### Socio-demographic characteristics

A total of 513 participants participated in this study with a response rate of 99%. Among the study participants, 305 (59.5%) were females. The mean age of the respondents was 36.11 years (SD ± 8.149 years). Most, 464 (90.4%), of the respondents were urban residents, and 435 (84.8%) were Amhara by ethnicity (Table 1).

**Table 1:** Socio-demographic and economic characteristics of HIV positive adults visiting ART clinic in Gondar University Referral Hospital, Northwest Ethiopia, 2015.

Characteristics		Frequency	Percent
**Sex**			
	Male	208	40.5
	Female	305	59.5
**Age**			
	18–29	101	19.7
	30–39	251	48.9
	≥40	161	31.4
**Residence**			
	Urban	464	90.4
	Rural	49	9.6
**Marital status**			
	Single	81	15.8
	Married	265	51.7
	Divorced	135	26.3
	Widowed	32	6.2
**Educational status**			
	No formal education	120	23.4
	Primary education	100	19.5
	Secondary education	244	47.6
	Higher education	49	9.5
**Religion**			
	Orthodox	443	86.4
	Muslim	55	10.7
	Protestant	15	2.9
**Ethnicity**			
	Amhara	435	84.8
	Tigre	28	5.5
	Others*	50	9.7
**Occupation**			
	Government employee	110	21.4
	House wife	107	20.9
	Merchant	99	19.3
	Private employee	88	17.2
	Farmer	38	7.4
	Others **	71	13.8
**Average monthly income**^a^			
	≤ 40	141	27.5
	40.05–75	153	29.8
	75.05–105	91	17.7
	>105	128	25
**No. of children**			
	Has no children	108	21.1
	1–3 children	330	64.3
	>3 children	75	14.6

*Quimant, Oromo;

**Daily laborer, student, retire, driver, living with family;

^a^Monthly income is in US Dollar

### Medical characteristics of respondents

The majority of the respondents, 274 (53.4%), knew their HIV status five years ago and the mean time since they tested HIV positive was 8.86 years (SD ± 2.8 years). Slightly more than a third, 204 (39.8%), of the study participants had CD4 count greater than 500/mm^3^, with the mean CD4 count of480.7/mm^3^ (SD ± 321.5/mm^3^). Most of the participants, 480(93.68%), had good clinical adherence.

### Partner related characteristics

Slightly more than a third, 358 (69.8%), of the respondents had sexual partners in the past three months preceding the data collection, and 49 (13.7%) had multiple sexual partners. Some, 87 (24.3%), of those who had sexual partners did not know their partner/s HIV sero-status and 28 (7.8%) had discordant result (Table 2).

**Table 2:** Partner related characteristics, partner's HIV status and disclosure among HIV positive adults visiting Gondar University Rreferral Hospital, Northwest Ethiopia, 2015.

Characteristics		Frequency	Percentage
Had sexual partner in the past three months (n = 513)			
	Yes	358	69.8
	No	155	30.2
Number of sexual partner/s in the past three months(n = 358)			
	One	309	86.3
	More than one	49	13.7
Type of sexual partner have in the past three months(n = 358)			
	Regular	291	81.3
	Non-regular	67	18.7
HIV status of their partner(n = 358)			
	Negative	28	7.8
	Positive	243	67.9
	Unknown	87	24.3
Discussion about safe sex with their partner/s(n = 358)			
	Yes	286	79.9
	No	72	20.1
Length of stay with current partner(n = 358)			
	<1year	74	20.7
	1-4Years	49	13.7
	>4year	235	65.6
HIV status disclosure to partner(n = 358)			
	Yes	275	76.8
	No	83	23.2

### Prevalence of risky sexual behaviors

**Fig 1:**
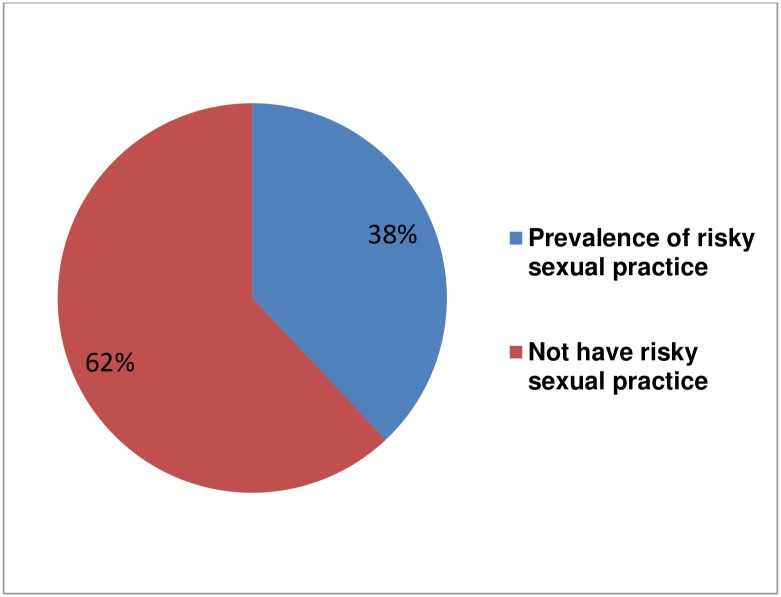
Prevalence of risky sexual practice among HIV positive adults attending Anti Retroviral Treatment clinics at Gondar University Referral Hospital, Northwest Ethiopia, 2015.

**Fig 2:**
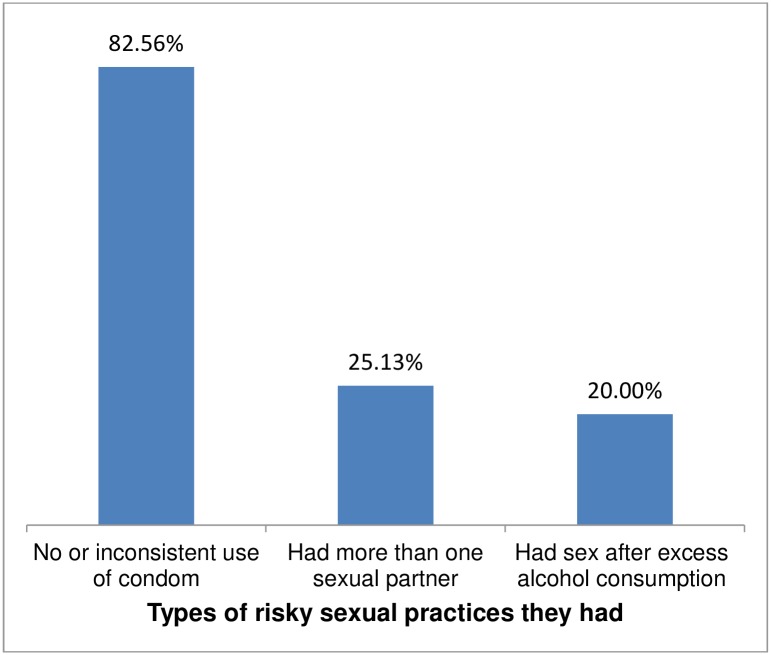
Frequency of different type of risky sexual practices among respondents who had risky sexual practices and attending Anti Retroviral Treatment (ART) clinic at Gondar University Referral Hospital, Northwest Ethiopia, 2015.

Among the study participants, 358(69.8%) were sexually active and 38% (95% CI: 33.3%, 42.3%) had risky sexual practice in the past three months preceding the date of data collection (Fig 1). Among those who had risky sexually practice, 161 (82.56%), did not use or inconsistently used condom during sexual contact, and 49 (25.13%) had sexual intercourse with multiple partners (Fig 2). Among the study participants who didn't use condom during sexual intercourse, 9.9% and 11.8% had multiple sexual partner and sex after excess alcohol consumption respectively. Among those who had sex after excess alcohol consumption, 69.23% had more than one sexual partner. The most common reasons mentioned for not using at all or inconsistently using condom are desire to have children, for 63(31.1%); partners refusal to use, for 37 (23%); fear to ask partner to use, for 24(14.9%); condom unavailability, for 18(11.2%); forgetfulness after alcohol intake, for 12(7.5%);religious prohibition use condom, for 10 (6.2%), and their perception that using condom has no more benefit for those who are already HIV infected, for 12(7.5%).

### Factors associated with risky sexual practice

Each explanatory variables was analyzed using bivariate logistic regression and variables having p-value less than 0.2 in bivariate analysis were fitted for multivariate logistic regression model in order to see the independent association between covariates and the outcome variable. In the multivariate analysis age, marital status, number of alive children they have, and CD4 count had statistically significant association with risky sexual practice.

Younger respondents had more risky sexual practices than older ones. The study participants whose age was in a range of 18–29 years were engaged in risky sexual practice 2.6 times (AOR = 2.63, 95% CI: 1.55, 4.47) higher than those whose age was greater or equal to 40 years. The odds of having risky sexual practice was 2 times (AOR = 2.29, 95% CI: 1.48, 3.53) higher in respondents whose age was in a range of 30–39 years as compared to those whose age was greater or equal to 40 years.

None married (single) study participants were 6 times (AOR = 6.32, 95%CI: 2.43, 16.44) more likely to engage in risky sexual practices than those who were widowed. Similarly, married HIV positive respondents were 6 times (AOR = 6.06, 95% CI: 2.81, 13.07) more likely to engage in risky sexual practice as compared to those who were widowed.

The study participants who had no child were 2.6 times (AOR = 2.58, 95% CI: 1.17, 5.72) more likely to engage in risky sexual practice than those who had greater than three children. The odds of having risky sexual practice were 2.6 times (AOR = 2.58, 95% CI: 1.57, 4.24) higher in the study participants whose CD4 count was greater than 500/mm^3^ as compared to those whose CD4 count was less than 350/mm^3^(Table 3).

**Table 3:** Bivariate and multivariate analyses of explanatory variables and risky sexual practice among HIV positive adults visiting ART clinic in Gondar University Referral Hospital, Northwest Ethiopia, 2015 (N = 513).

Variables		Risk Sexual Practice		COR(95%CI)	AOR(95% CI)
		Yes	No		
Age					
	18–29	47	54	8.56 (95%CI:2.55–16.68)	2.63 (95%CI:1.55–4.47)*
	30–39	108	143	4.82 (95%CI:1.39–6.68)	2.29 (95%CI:1.48–3.53)*
	≥40	40	121	1	1
Marital Status					
	Single	44	37	9.65 (95%CI:4.25–21.88)	6.32 (95%CI:2.43–16.44)**
	Married	114	151	6.12 (95%CI:2.94–12.77)	6.06 (95%CI:2.81–13.07)**
	Divorced	28	57	0.67. (95%CI:0.44–1.13)	0.58 (95%CI:0.41–1.62)
	Widowed	9	73	1	1
Residence					
	Urban	175	289	1	
	Rural	20	29	1.14(95%CI:1.04–2.08)	
Monthly Income^a^					
	≤ 40	37	104	0.44(95%CI: 0.27–0.74)	
	40.05–75	63	90	0.87 (95%CI:0.54–1.40)	
	75.05–105	38	53	0.89(95%CI:0.52–1.54)	
	>105	57	71	-1	
Number of alive Children					
	No children	58	50	2.98 (95%CI:1.59–5.60)	2.58(95%CI:1.17–5.72)*
	1–3	116	214	1.39(95%CI:0.80–2.42)	1.32(95%CI:0.73–2.39)
	> 3	21	54	1	1
Clinical Adherence					
	Good	175	304	1	
	Poor	20	14	2.48(95%CI:1.22–5.04)	
CD4 Count					
	<350/mm^3^	34	90	1	1
	350-500/mm^3^	40	98	1.08(95%CI:0.63–1.85)	1.05(95%CI: 0.59–1.85)
	>500/ mm^3^	121	130	2.46(95%CI:1.55–3.93)	2.58(95%CI:1.57–4.24)**

Note: 1 = reference.

^a^ Monthly income is in US Dollar,

*P-value is between the range 0.05 to 0.01,

**P-value less than 0.01, Hosemer Lemshow Test = 0.768

## Discussion

It is highly recommended that HIV infected people should maintain safe sex practice so as not to transmit the disease to HIV free individuals or not to transfer or acquire new strain of HIV between HIV positive concordant partners. Thus, unprotected/unsafe sex, having multiple sexual partners and other risk behaviours in people living with HIV/AIDS are important areas of concern because of the risk of contracting and /or transmitting the virus.

In this study, risky sexual practice is defined as having one or more of the following practices during the past three months prior to date of data collection: having multiple sexual partners, casual sex, sex without or inconsistent use of condom even with regular partner, sex with the influence of substance like alcohol.

A substantial number of HIV positive clients, who were attending care at Gondar University Referral Hospital were engaged in risky sexual practice. In this study, more than a third, 38% (95% CI: 33.3%, 42.3%), of the study participants had risky sexual practice in the past three months preceding data collection. This finding is consistent with studies conducted in Addis Ababa (36.9%)[8], (30.4%)[11], South Africa, Johannesburg (34.0%)[12], and Togo (34.6%)[13]. However, the finding is higher than those of studies done in Debrezeit, Ethiopia (22.2%)[5], and Kenya (28%)[14]. The possible reason for this variation might be difference in the length of time used to measure the prevalence of risky sexual practice. For example, in the Debrezeit study, one month preceding the date of data collection was considered. Another possible reason can be the difference in measurements that is only inconsistent condom use was considered to define risky sexual practice in these studies.

The prevalence of risky sexual practice in this study was lower than that of studies conducted in Kumasi, Ghana (51%)[15] and Nigeria (56%)[16]. This difference might be due to variations in the study settings that is the socio-demographic and economic characteristics of the study participants. There might also be difference in educational levels. Hence, low economic and educational status can predispose people to engage in risky sexual practices.

The chance to engage in risky sexual practice is higher in young individuals than old ones because of the following possible reasons: young individuals are usually sexually hyperactive; sexual act in younger individuals is usually unplanned; they are less likely to control their impulsive sexual desire; they commonly take alcohol, and most of the time they are not married. Thus, they have more chance to have casual sex and/or multiple sexual partners. In this study, HIV positive individuals who were in the age range of 18–39 years had more risky sexual practices than those whose age was greater or equal to forty years. A study in north-western Ethiopia noted that being aged had less risk for unsafe/unprotected sex[17]. The same finding was observed in a study done in Dar Es Salaam, Tanzania[18].

None married individuals are usually young and may have multiple sexual partners. Hence, they are more prone to have risky sexual practice. In this study, it is noted that none married individuals were six times more likely to engage in risky sexual practices than widowed ones. Accordingly, married individuals were six times more likely to have risky sexual practice than widowed clients. This might be due to the fact that widowed individuals might be at an advanced clinical stage of the disease which might in turn reduce sexual desire. Furthermore, HIV concordant partners might think that the condom is no more useful for HIV prevention. A similar finding was observed in a study conducted in Addis Ababa[11]. A study conducted in Uganda also noted that there was low level of abstinence among young and married participants[19].

The increased accessibility of the prevention of mother to child transmission (PMTCT) of HIV services and the improved community understanding of the high probability of getting an HIV free child supported by the intention of couples who have no children may lead to the avoidance of the condom. In addition, individuals who have no children are usually younger and relatively sexually hyperactive. Thus, they are prone to engage in risky sexual practices. In this study, it was found that the odds of engaging in risky sexual practice was three times higher in those who had no children as compared to those who had more than three children. A study conducted in Uganda identified that there was a strong association between condom use (safe sex) and no desire for more child among people living with HIV[19].

When the CD4 count of HIV positive individuals improves, which can be because of the effect of the antiretroviral therapy (ART), such individuals may resume to sexual activity which may take part for increased risky sexual practice. In another hand, when the immunity of individuals is decreased, their clinical condition deteriorates. Thus, they become more likely to abstain from sexual practice. In this study, it has been identified that HIV positive individuals whose CD4 count were more than 500/mm^3^ had more risky sexual practice than those whose CD4 count were less than 350/mm^3^.

The possible limitation of this study could be its using interviewer administered data collection technique which might result in a social desirability bias because of the sensitive nature of the issue. This might lead to under estimating of the prevalence of risky sexual practice. However, attempts were made to inform respondents about the importance of providing the real/actual data.

## Conclusions

The prevalence of risky sexual practice in the past three months among HIV positive individuals who attended the chronic care and treatment clinic at Gondar University Hospital was high. This indicates that a considerable number of clients continued to transmit HIV or transmit or acquire new viral strains which may attribute for drug resistant infections. Younger individuals, both married and unmarried, clients who had no child, and those whose immunity was intact (better CD4 count) were important predictors of risky sexual practice. So, it is strongly recommended that the Regional Health Bureau and health service providers working at Gondar University Hospital, especially in the ART clinic need to work hard on behavioral change communication. Specifically, health care providers need to have regular and ongoing counseling sessions as a part of the routine ART services. The health bureau has also a responsibility to design a tailored strategies and to support and monitor health facilities and health service providers for its implementation.

## Supporting information

S1 File Consent form & questionnaire- English version. (DOCX)

S2 File Consent form & questionnaire- Amharic version. (DOCX)

## Abbreviations

AIDS: Acquired Immuno Deficiency Syndrome
AOR: Adjusted odds ratio
ART: Anti Retroviral treatment
COR: Crud Odds Ratio
ETB: Ethiopian Birr
HAART: Highly Active Anti-Retroviral Treatment
HIV: Human Immunodeficiency Virus
PLWHA: People Living With HIV/AIDS
SSA: Sub Saharan Africa
STIs: Sexually Transmitted Infections
WHO: World Health Organization
