# Supplementary material for: Risky sexual practice and associated factors among HIV positive adults attending anti-retroviral treatment clinic at Gondar University Referral Hospital, Northwest Ethiopia
Source: PLoS One. 2017 Mar 28;12(3):e0174267. doi: 10.1371/journal.pone.0174267 (PMC5369687; doi:10.1371/journal.pone.0174267)
Supplement: S1 File — (DOCX) [file pone.0174267.s001.docx]

## Annex 2: Consent form and questionnaire

Hello! How are you? My name is _____________. I am here on the behalf of investigators who are working in University of Gondar, College of medicine and health science in order to collect information related to risky sexual behavior from clients receiving ART. So, this is a questionnaire prepared to assess sexual behavior of people living with HIV/AIDS who are on ART in Gondar university Hospital. The main aim of the study is to provide basic information about the sexual behavior of people living with HIV/AIDS who are on ART. The correct information that you are going to provide us has great importance to set an effective preventive and interventional strategy for HIV/AIDS in the country. There is no any risk that you will face by participating in this research except devoting your time to respond to the interview. I strongly assure that your name and other identification will not be documented in the questionnaire and the information you going to provide us will be kept confidential and will not be used for anything other than research purpose. You are not forced to participate and you have the right not to participate or to quit at any time in between. But we encourage you to respond to the questions and give us the required information. The question will not take more than 30 minutes. You can ask any question at any time.

Having this information, are you willing to participate in the study?

Agree Disagree

If the client agrees, thank him/her and proceed to the interview.

If the client disagrees, thank him/her and proceed to the next eligible client.

**Questionnaire (English version)**

**Part I**. **Socio-demographic information**

| No | Questions | Response | Remark |
| --- | --- | --- | --- |
| 101 | Sex | 1. Male 2. Female |  |
| 102 | Age (in completed year) | ________ |  |
| 103 | Residence | 1. Urban 2. Rural |  |
| 104 | Marital status | 1. Single 2. Married 3. Divorce 4. Widowed |  |
| 105 | Educational status | 1. No formal education 2. primary 3. secondary 4. college/university |  |
| 106 | Religion | 1. Orthodox Christian 2. Protestant 3. Muslim 4. Catholic 5. Other (specify)________ |  |
| 107 | Ethnicity | 1. Amhara 2. Tigre 3. Oromo 4. other (specify)__________ |  |
| 108. | Occupation | 1. Government employee 2. Private employee 3. Housewife 4. Commercial sex worker 5. Merchant 6. Farmer 7. Drive 8. Other specify)________ |  |
| 109 | Average monthly income | _________Eth birr |  |
| 110 | How many children do you have | _____________________ |  |

**Part II Partner related characteristics**

| S.no. | Questions | Response | Remark |
| --- | --- | --- | --- |
| 201 | Do you have any partner in the past three months? | 1. Yes 2. No | If no skip to Q 301 |
| 202 | How many partner(s) do you have in the past three months? | 1. One  2. More than one |  |
| 203 | Educational status of partner | 1. No formal education 2. Primary school 3. Secondary school 4. college/university 5. Unknown |  |
| 204 | Partner’s occupation | 1. Government employee  2. Housewife  3. Merchant  4. Private employee  5. Farmer  6. Other(specify)________ |  |
| 205 | Type of partner(s) with whom you had sex in the past three months? | 1. Regular  2. Commercial partner  3. Non-regular partner | More than one answer is possible |
| 206 | Do you know the HIV status of the partner(s) with whom you had sex in the past three months? | 1. Yes 2. No |  |
| 207 | If yes to Q. 206 what was/were the HIV status of the partner/s with whom you had sex in the past three months? | 1. Negative 2. Positive |  |
| 208 | Have you discussed about safe sex with your partner(s)? | 1. Yes 2. No |  |
| 209 | Length of stay with current partner | _________year(s) |  |
| 210 | Have you disclose your HIV status to your sexual partner(s)? | 1. Yes  2. No |  |

**Part III** **Medical related factors**

| No | Questions | Possible Responses | Remark |
| --- | --- | --- | --- |
| 301 | How long is it since you tested HIV positive? | _______year |  |
| 302 | Have you started ART? | 1. Yes 2. No | If no, skip to Q 305 |
| 303 | If yes how long is it since you have started ART? | ________( in years) |  |
| 304 | Level of drug adherence | 1. Good 2. Fair 3. Poor | Fill this by reviewing patients' document |
| 305 | Level of clinical adherence | 1. Poor 2. Good | Fill this by reviewing patients' document |
| 306 | Patient's current/recent CD4 count? | _________/mm^3^ | Fill this by reviewing patients' document |

**Part IV: Knowledge/attitude related questions**

| No | Questions | Possible Responses | Remark |
| --- | --- | --- | --- |
| 401 | Do you think having sex after excess alcohol consumption is risky? | 1. Yes 2. No |  |
| 402 | Do you believe that unprotected sex between HIV positive partners is risky for the disease progression? | 1. Yes  2. No |  |
| 403 | Do you believe that using condom is important during sexual act between HIV positive partners? | 1. Yes 2. No |  |
| 404 | Do you believe that having multiple sexual partners is risky for HIV transmission? | 1. Yes 2. No |  |
| 405 | Do you believe that disclosing self HIV status to partner/s is important to prevent disease transmission? | 1. Yes 2. No |  |
| 406 | Do you believe that getting infected with other sexually transmitted disease will enhance HIV transmission? | 1. Yes 2. No |  |
| 407 | Do you think taking HAART reduce HIV transmission? | 1. Yes 2. No |  |

**Part V: Behavioral factors**

| **No** | **Question** | **Response** | **Remark** |
| --- | --- | --- | --- |
| 501 | Have you used any substance in the last one year? | 1. Yes 2. No |  |
| 502 | If yes to Q. 501 which type(s) have you used? | 1. Chat 2. Cigarette 3. Alcohol 4. Hashish 5. Shisha 6. Others(specify)________ |  |
| 503 | Do you have any sexual intercourse in the past three months? | 1. Yes 2. No | If no, stop the interview here |
| 504 | If yes, have you had sex after substance use? | 1. Yes 2. No |  |
| 505 | Have you had sex with the influence of alcohol? | 1. Yes 2. No |  |
| 506 | Have you disclosed your HIV status to your partner/s with whom you had sexual contact? | 1. Yes 2. No |  |
| 507 | Have you had sex with more than one partner? | 1. Yes 2. No |  |
| 508 | Have you used condom during sexual intercourse? | 1. Yes 2. No |  |
| 509 | If yes to Q 508, how often you have used condom in the past three months? | 1. Always 2. Most of the time 3. Sometimes |  |
| `510 | If your answer for Q.508 is and for Q.509 is 2 or 3, what was/were the reason(s) for not using condom consistently/always? | 1. My partner did not want to use condom 2. My partner already HIV positive 3. Condom was not available 4. I fear to ask my partner to use a condom 5. Thinking that ART prevents the acquisition and transmission of HIV 6. Was drank and didn’t think of condom use 7. Wanted to have a child (own/partner) 8. Condom is against my religion 9. I didn’t use because I am already infected 10. Other(specify)________ | (Multiple responses are possible) |

Thank the respondent,

Interviewer signature __________________ Date ______/______/________
