# Supplementary material for: Risky sexual practice and associated factors among HIV positive adults attending anti-retroviral treatment clinic at Gondar University Referral Hospital, Northwest Ethiopia
Source: PLoS One. 2017 Mar 28;12(3):e0174267. doi: 10.1371/journal.pone.0174267 (PMC5369687; doi:10.1371/journal.pone.0174267)
Supplement: S2 File — (DOCX) [file pone.0174267.s002.docx]

**የፈቃድ መጠየቂያ ቅፅ**

ጤና ይስጥልኝ ስሜ__________________እባላለሁ፡፡ እዚህ የተገኘሁት በጎንደር ዩኒቨርሲቲ በህ/ጤ/ሳ/ኮሌጅ ሰራተኖችና ተመራማሪዎችን ወክየ የፀረ-ኤች.አይ.ቪ መድሃኒትና እንክብካቤ ተጠቃሚ ከሆኑ ደንበኞች ጥንቃቄ የጎደለ የግብረ ስጋ ግንኙነትና ተያያዥ ባህሪያትን በተመለከተ ጥናት ለማድረግ መረጃ ለመሰብሰብ ነዉ፡፡ የጥናቱ ዋና አላማ አጋላጭ የሆኑ የግብረ ስጋ ግንኙነት ባህሪያት ላይ መሰረታዊ የሆነ መረጃን ለመስጠት ነዉ፡፡ ከእርስዎና ከሌሎች ድንበኞች የሚገኘዉ ትክክለኛ መረጃ ለደንበኞች የምንሰጠዉን አገልግሎት ለማስተካከልና ለማሻሻል እንዲሁም በአገሪቱ ኤች.አይ.ቪ. ኤድስን ለመከላከልና ለማከም ዉጤታማ የሆኑ አቅጣጫዎችን ለማስቀመጥ ከፍተኛ የሆነ ጥቅም ይኖረዋል፡፡ ጥያቄዎቹን ለመመለስ ከሚወስደው ሰዓት ወይም ጊዜ ዉጭ በጥናቱ በመሳተፍዎ ሊደርስብዎ የሚችል ምንም ዓይነት ጉዳትም ሆነ ችግር የለም፡፡ በሚሰጡን መረጃ ላይ ስምም ሆነ ሌላ እርስዎን ሊገልፅ የሚችል ነገር እንደማይቀመጥና መረጃዉም በሚስጥር እንደሚያዝና ከዚህ ጥናት ዉጭ ለምንም ዓይነት አገልግሎት እንደማይዉል በጥብቅ አረጋግጥልዎታለሁ፡፡ በጥናቱ እናዲሳተፉ አይገደዱም እንዲሁም በማንኛዉም ሰዓት ጥናቱን አቋርጠዉ የመዉጣት መብት አለወት ፡፡ነገር ግን በጥናቱ እንዲሳተፉና አስፈላጊ የሆኑ መረጃዎችን እንዲሰጡን እንጠይቃለን፡፡ ጥያቄዉ ከሰላሳ ደቂቃ በላይ አይወስድም፤ ማንኛዉንም ዓይነት ጥያቄ በማንኛዉም ሰዓት መጠየቅ ይቻላል፡፡

ተመራማሪዉ ስለጥናቱ ዓለማ ሁሉንም መረጃዎች ገልፆልኛል በተጨማሪም በጥናቱ ያለመሳተፍና ተሳትፎየንም በማንኛዉም ሰዓት የማቋረጥ መብት እንዳለኝ ተገንፆልኛል ፡፡

በዚህ መረጃ መሰረት በጥናቱ ለመሳተፍና አስፈላጊዉን መረጃ ለመስጠት ተስማምተዋል?

ተስማምቻለሁ አልተስማማሁም

ደንበኛዎ በጥናቱ ለመሳተፍ ፍቃደኛ ከሆኑ አመስግነው መረጃ መሰብሰብዎን ይጀምሩ፡

ደንበኛዎ በጥናቱ ለመሳተፍ ፍቃደኛ ካልሆኑ አመስግነው ወደ ሌላ ደንበኛ ይሂዱ፡፡

የመረጃ መሰብሰቢያ ቅፅ (በአማርኛ)

**ክፍል አንድ** ፡ አጠቃላይ መረጃ

| **ተ.ቁ** | **ጥያቄ** | **መልስ** | **ምርመራ** |
| --- | --- | --- | --- |
| 101 | ፆታ | 1. ወንድ 2. ሴት |  |
| 102 | እድሜ | ___________________________ |  |
| 103 | የመኖሪያ አደራሻ | 1. ከተማ 2. ገጠር |  |
| 104 | የጋብቻ ሁኔታ | 1. ያላገባ/ች 2. ያገባ/ች 3. የተፋታ/ች 4. የሞተበት/ባት |  |
| 105 | የትምህርት ደረጃ | 1. መደበኛ ት/ት ያልተከታተሉ 2. አንደኛ ደረጃ 3. ሁለተኛ ደረጃ 4. ዲፕሎማ ከዚያ በላይ |  |
| 106 | ሀይማኖት | 1. ኦርቶዶክስ ክርስትያን 2. ፕሮቴስታንት 3. ሙሰሊም 4. ካቶሊክ 5. ሌላ ካለ________ |  |
| 107 | ብሄር | 1. አማራ 2. ትግሬ 3. ኦሮሞ 4. ሌላ __________ |  |
| 108 | ስራ | 1. የመንግስት ሰራተኛ 2. የግል ተቀጣሪ 3. የቤት እመቤት 4. ሴተኛ አዳሪ 5. ነጋዴ 6. ገበሬ 7. ሹፌር 8. ሌላ ካለ( ይጠቀስ)________ |  |
| 109 | አማካኝ የወር ገቢ | _________ የኢትዮጵያ ብር |  |
| 110 | ስንት ልጅ አለዎት | _________________________ |  |

**ክፍል ሁለት** ፡ **የትዳር አጋርን ፤ ወንድ/ ሴት ጓደኛን የሚመለከቱ ጥያቄዎች**

| **ተ.ቁ** | **ጥያቄ** | **መልስ** | **አስተያየት** |
| --- | --- | --- | --- |
| **201** | ባለፈዉ ሦስት ወራት የግብረ-ስጋ ግንኙነት አድርገው ነበር? | 1. አወ 2. አላደረግሁም | አላደረግሁም ካሉ ወደ ተ.ቁ 301 ይሂዱ |
| **202** | መልስወ አወ ከሆነ በዚህ ሦስት ወራት ከስንት ሰዉ ጋር ግብረ-ስጋ ግንኙነት ነበረዎት? | 1. ከአንድ ሰዉ ጋር 2. ከአንድ ሰዉ በላይ |  |
| **203** | የትዳር አጋርዎ ፤ የወንድ/ሴት ጓደኛዎ የትምህርት ደረጃ? | 1. መደበኛ ት/ት ያልተከታተሉ 2. አንደኛ ደረጃ 3. ሁለተኛ ደረጃ 4. ዲፕሎማ ከዚያ በላይ 5. አላውቅም |  |
| **204** | የትዳር አጋርዎ ወይም የወንድ/ሴት ጓደኛዎ ስራ? | 1. የመንግስት ሰራተኛ 2. የቤት እመቤት 3. የግል ተቀጣሪ 4. ነጋዴ 5. ገበሬ 6. ሌላ ካለ(ይጠቀስ)_______ |  |
| **205** | በባለፈዉ ሦስት ወራት የግብረ ስጋ ግንኙነት የፈጸሙት ከማን ጋር ነው? | 1. ከባለቤቴ/ከቋሚ ጓደኛየ ጋር 2. ከሴተኛ አዳሪ ጋር 3. ባል/ሚስት ወይም ቋሚ ጓደኛ ካልሆነ ሰዉ ጋር | (ከአንድ በላይ መመለስ ይቻላል) |
| **206** | ግብረ-ስጋ ግንኙነት የፈፀሙበት ሰዉ የኤች.አይ.ቪ. ሁኔታ ምን እንደ ነበር ያዉቃሉ? | 1. አወ 2. አላዉቅም |  |
| **207** | ለጥያቄ ቁጥር **206** መልስወ አወ ከሆነ የኤች.አይ.ቪ. ሁኔታዋ/ው ምንድን ነዉ? | 1. ነፃ 2. ቫይረሱ በደሙ/ሟ ዉስጥ ያለ |  |
| **208** | ጥንቃቄ ስለተሞላበት ግብረ-ስጋ ግንኙነት ከትዳር አጋርዎ ፤ ከወንድ ወይም ከሴት ጓደኛዎ ጋር ዉይይት አድርገው ያዉቃሉ? | 1. አወ 2. አላዉቅም |  |
| **209** | ከአሁኑ ጓደኛዎ ጋር ለምን ያክል ጊዜ አብረው ቆይተዋል? | ___________ዓመት |  |
| **210** | የኤች. አይ. ቪ ዉጤትዎን ለባለቤትዎ ወይም ወንድ/ ሴት ጓደኛዎ ግልፅ አድርገዋል? | 1. አወ 2. አደለም |  |

**ክፍል ሶስት. ከህክምና ጋር የተገናኙ ጥያቄዎች**

| **ተ.ቁ** | **ጥያቄ** | **መልስ** | **አስተያየት** |
| --- | --- | --- | --- |
| 301 | ኤች.አይ.ቪ በደምዎ ዉስጥ ከተገኘ ምን ያክል ጊዜ ይሆነዋል? | __________ዓመት |  |
| 302 | የፀረ-ኤች.አይ.ቪ መድኃኒት ጀምረዋል? | 1. አወ 2. አልጀመርኩም | መልስዎ አልጀመርኩም ከሆነ ወደ ጥያቄ ቁጥር 305 ይሂዱ |
| 303 | ለጥያቄ ቁጥር 302 መልስዎ አዎ ከሆነ ከጀመሩ ምን ያክል ጊዜ ይሆነዎታል? | ________(አመት) |  |
| 304 | የመድሃኒት ቁርኝት ሁኔታዉ | 1. ከፍተኛ 2. መጠነኛ 3. ዝቅተኛ | የበሽተኛዉን ካርድ በማየት የሚሞላ |
| 305 | የህክምና ክትትል ቁርኝት ሁኔታ አንዴት ነው | 1. ከፍተኛ 2. ዝቅተኛ | የበሽተኛዉን ካርድ በማየት የሚሞላ |
| 306 | የበሽተኛዉ የወቅቱ/የቅርብ ጊዜ ሲዲ4 መጠን | ________ | የበሽተኛዉን ካርድ በማየት የሚሞላ |

**ክፍል አራት:** **ጥንቃቄ ስለጎደለዉ ግብረ ስጋ ግንኙነት እዉቀትን የሚሚዳስሱ ጥያቄዎች :**

| **ተ.ቁ** | **ጥያቄ** | **መልስ** | **አስተያየት** |
| --- | --- | --- | --- |
| **401** | ከመጠን በላይ አልኮል ጠትቶ ግብረ-ስጋ ግንኙነት ማድረግ ለኤች.አይ፣ቪ ያጋልጣል ብለዉ ያስባሉ? | 1. አወ 2. የለም |  |
| **402** | በሁለት ቫይረሱ በደማቸዉ ዉስጥ ባለባቸዉ ሰወች መካከል የሚደረግ ጥንቃቄ የጎደለዉ ግብረ-ስጋ ግንኙነት ለበሽታዉ መባባስ ምክንያት ነዉ ብለዉ ያስባሉ? | 1. አወ 2. የለም |  |
| **403** | ቫይረሱ በደማቸዉ ዉስጥ ባለባቸዉ ሰወች መካከል በሚደረግ ግብረ-ስጋ ግንኙነት ወቅት ኮንዶም መጠቀም አስፈላጊ ነዉ ብለዉ ያስባሉ? | 1. አወ 2. የለም |  |
| **404** | ከአንድ በላይ ከሆነ ሰዉ ጋር የግብረ ስጋ ግንኙነት ማድረግ ለኤች.አይ.ቪ መተላለፍ ምክንያት ይሆናል ብለዉ ያስባሉ? | 1. አወ 2. የለም |  |
| **405** | የኤች.አይ.ቪ ዉጤትዎን ለባለቤትዎ / ወንድ ወይም ሴት ጓደኛዎ ግልፅ ማድረግ ኤች.አይ.ቪን ለመከላከል አስፈላጊ ነዉ ብለዉ ያስባሉ? | 1. አወ 2. የለም |  |
| **406** | በሌላ በአባለዘር በሽታ መያዝ በኤች.አይ.ቪ መያዝን ወይም ማስተላለፍን ይጨምራል ብለዉ ብለዉ ያስባሉ? | 1. አወ 2. የለም |  |
| **407** | የፀረ-ኤች.አይ.ቪ መድሃኒት የኤች.አይ.ቪ ስርጭትን /መተላለፍን ይከላከላል ብለዉ ያስባሉ? | 1. አወ  2. የለም |  |

**ክፍል አምስት** : **የግል ባህሪን የሚመለከቱ ጥያቄዎች**

| ተ.ቁ | **ጥያቄ** | **መልስ** | **አስተያየት** |
| --- | --- | --- | --- |
| 501 | ባለፈዉ አንድ ዓመት ዕፅ ተጠቅመዉ ያዉቃሉ? | 1. አወ 2. የለም |  |
| 502 | መልስዎ አወ ከሆነ ምን ዓይነት ዕጽ ነዉ የተጠቀሙት? | 1. ጫት 2. የአልኮል መጠጥ 3. ሲጋራ( ትምባሆ) 4. ሀሽሽ 5. ሺሻ 6. ሌላ ካለ (ይጠቀስ)_____________ |  |
| 503 | ባለፈዉ ሶስት ወራት የግብረ -ስጋ ግንኙነት አድርገዋል? | 1. አወ 2. አልፈፀምኩም | አልፈፀምኩም ካሉ ይህ የቃለመጠየቁ መጨረሣ ይሆናል |
| 504 | መልሱ አወ ከሆነ አደንዛዥ ዕፅ ከተጠቀሙ በኋላ የግብረ -ስጋ ግንኙነት ፈፅመዉ ያዉቃሉ? | 1. አወ 2. አላዉቅም |  |
| 505 | አልኮል ከመጠን በላይ ጠጥተዉ የግብረ -ስጋ ግንኙነት ፈፅመዉ ያዉቃሉ? | 1. አዎ 2. አላዉቅም |  |
| 506 | ፆታዊ ግንኙነት ሲፈፅሙ ኤች.አይ.ቪ በደምዎ ዉስጥ እንዳለ ግልፅ አድርገዋል? | **1.** አወ  **2**. አላደርግም |  |
| 507 | የግብረ-ስጋ ግንኙነት ነበረወትከአንድ ሰዉ በላይ ነው? | **1**. አወ  **2**. የለኝም |  |
| 508 | የግብረ-ስጋ ግንኙነት ሲፈፅሙ ኮንዶም ተቅመዋል? | 1. አወ 2. አልጠቀምም |  |
| 509 | ለጥያቄ ቁጥር **508** መልስዎ አወ ከሆነ ኮንዶምን ምን ያክል ጊዜ ይጠቀማሉ? | 1. ሁል ጊዜ 2. አብዛኛዉን ጊዜ 3. አልፎ አልፎ |  |
| 510 | ለጥያቄ ቁጥር 508 መልስዎ 2, ከሆነ ወይም ለጥያቄ ቁጥር 509 መልስወ 2 ወይም 3 ከሆነ ኮንዶምን ሁልጊዜ ላለመጠቀምወ ምክንያቱ ምን ነበር? | 1. ፆታዊ ግንኙነት የፈፀምኩበት ሰዉ ኮንዶም መጠቀም ስለማይፈልግ 2. ፆታዊ ግንኙነት የፈፀምኩበት ሰዉ ኤች.አይ.ቪ ስላለበት 3. ኮንዶም ስላልነበረ 4. ኮንዶም እንዲጠቀም/ እንድትጠቀም መጠየቅ ስለፈራሁ 5. ኤ.አር.ቲ መጀመር ቫይረሱ እዳይተላለፍ ስለሚያደረግ 6. አልኮል ጠጥቸ ስለነበር ኮንዶም ስለመጠቀም አላሰብኩም 7. ልጅ መዉለድ ስለፈለኩ/ ስለፈለግን 8. ኮንዶም መጠቀምን ሀይማኖቴ ስለማይፈቅድ 9. አንድ ጊዜ በቫይረሱ ተይዣለሁ ብየ ስላሰብኩ 10. ሌላ ካለ ይጠቀስ_____________ | (ከአንድ በላይ መመለስ ይቻላል) |

በመጨረሻ ደንበኛህን አመስግነህ ተሰናበት

የመረጃ ሰብሳቢው ፊርማ __________________ ቀን______/______/________
